# Healthcare costs for the elderly in Japan: Analysis of medical care and long-term care claim records

**DOI:** 10.1371/journal.pone.0190392

**Published:** 2018-05-14

**Authors:** Naomi Akiyama, Takeru Shiroiwa, Takashi Fukuda, Sachiyo Murashima, Kenshi Hayashida

**Affiliations:** 1 Iwate Medical University, School of Nursing, Iwate, Japan; 2 Department of Health and Welfare Services, National Institute of Public Health, Wako, Saitama, Japan; 3 Nursing and Health Sciences, Oita University, Oita, Oita, Japan; 4 Department of Medical Informatics and Management, University Hospital, University of Occupational and Environmental Health, Kitakyushu, Fukuoka, Japan; Public Library of Science, UNITED KINGDOM

## Abstract

**Background:**

The population is aging rapidly in many developed countries. Such countries need to respond to the growing demand and expanding costs of healthcare (HC) for the elderly. Therefore, it is important to investigate the factors correlating such HC costs. In Japan, HC is composed of two sections, namely medical care (MC) and long-term care (LTC). While many studies have examined MC and LTC costs on their own, few studies have conducted comprehensive investigations of HC costs. The aim of this study is to examine the risk factors that influence HC costs for the elderly who enroll in the LTC insurance system in Japan.

**Methods:**

The inclusion criteria in the present study are as follows: being 65 years of age, or older; certified eligibility for, and use of services offered by the LTC insurance system at home or in an institutional setting in December 2009; and being covered by the National Health Insurance (NHI) system. MC and LTC insurance data were obtained from claim records for the elderly in July and December of 2007, 2008, and 2009 (i.e., a total of six survey points). Panel data, per subject, were constructed using MC and LTC claim records. The sample included 810 subjects and 4029 observations.

**Results:**

We estimated a regression equation with a censored dependent variable using a Tobit model. Significant associations between MC or LTC costs and interaction terms (household composition × seasonal effects) were investigated. MC costs significantly decreased and LTC costs significantly increased among subjects living alone during winter. Income level was also a positive determinant of MC costs, while eligibility level was a positive determinant of LTC costs.

**Conclusions:**

We recommend that the health policy for the elderly focus more on seasonal effects, household composition, and income level, as well as on eligibility level.

## Introduction

According to the Organization for Economic Cooperation and Development (OECD), many developed countries will experience an aging society as the baby boom generation ages. Consequently, the demand for long-term care (LTC) is expected to rise sharply over the coming three decades [1]. Such countries need to respond to the growing demand for, and cost of LTC.

Japan, in particular, is at the forefront of countries experiencing an aging society. The healthcare (HC) policy in Japan includes both medical care (MC) and LTC insurance systems. MC insurance is necessary for medical treatment due to illness, injury, preparation for hospitalization, surgery, outpatient visits, and medication treatment. LTC insurance applies when a person requires nursing care. The MC insurance service covers hospitalization, rehabilitation, medication, medical treatment, surgery, and visiting nursing services. The LTC insurance service covers in-home care and community-based care, such as residential facilities and facility care. If an insured person is over the age of 65, he/she is eligible for the MC insurance service and the LTC insurance service. If the insured person needs acute medical care, he/she will be admitted to a hospital and will receive medical treatment. After being discharged, the person can use in-home care services, including housekeeping and nursing care, on a daily basis, and can receive rehabilitation services in order to maintain his/her physical activity ability, which does not fall under acute treatment rehabilitation. These are services provided by LTC insurance. However, the two insurance services are separate and provided independently. Since 1961, Japan has provided a universal National Health Insurance (NHI) system for MC to all its citizens. The LTC insurance system, a mandatory social system supporting LTC for the elderly, was established in 2000 [2]. The system encompasses a wide variety of services for elderly individuals who require assistance in performing basic activities in their daily lives. In 2010, the total expenditure for MC insurance reached USD 458 billion (USD 1 = JPY 80 in 2011), of which USD 203 billion was spent on the elderly. The total expenditure for LTC insurance grew from USD 45 billion in 2000 to USD 99 billion in 2010 [3]. HC costs are the sum of MC costs and LTC costs. The rapid increase in HC costs has cast doubt over the sustainability of the MC and LTC insurance systems in Japan. Nevertheless, HC expenditure in Japan is relatively low compared to that of the United States and other OECD member countries [4]. With a history of having a healthy population and low-cost HC, the priority for Japanese health policy should be on improving access to affordable HC and placing greater emphasis on the efficiency and quality of HC services.

A behavioral model proposed by Andersen [5, 6] and Andersen and Newman [7] is frequently employed to analyze factors associated with HC services. The framework of this model has broad applicability and enables analyses across age, country, and various segments of society [8]. The model includes four interacting variables: environment, population characteristics, health behavior, and outcomes. Many previous studies have focused on household composition, which Andersen’s model includes among the population characteristics used as independent variables. The proportion of the elderly living alone has increased in most OECD countries, and the demand for HC services among individuals living alone will continue to increase steadily [1]. Several studies have reported that household composition is related to HC services. For example, it has been suggested that the use of LTC services among elderly persons living alone is higher than among those living with others [9]. According to a recent report, the number of Japanese aged 65 or older who live alone was 4.98 million in 2010, and this is expected to increase by 53%, to approximately 7.62 million, in 2035 [10]. Among the independent variables included in Andersen’s model, we also focus on environmental variables. Phillips et al. [11] conducted a systematic review of the literature on HC services, and found that environmental variables were the most common variables included in this literature. These include an urban or rural location, region of admission to hospital or LTC facilities, and the supply of services [11, 12].

Many studies have reported that seasons have a significant impact on health. For example, studies have reported that institutionalization rates peak in winter [13], as does the demand for intensive care units (ICUs) [14]. Increased winter mortality in ICUs has also been reported [15]. In addition, the elderly are more likely to be affected by hot or cold weather. For example, a study reported that the incidence of the hip joint fractures increased from spring to winter [16]. We hypothesize that MC costs and LTC costs are influenced by the environmental variable, seasonal effects. This tendency may be stronger when seasonal effects interact with household composition. Therefore, this study examines two research questions: (1) Are MC costs and LTC costs influenced by seasonal effects among Japanese people aged 65 or older? (2) Are MC costs and LTC costs influenced strongly by the interaction between seasonal effects and household composition?

## Materials and methods

### Study setting

A longitudinal design was applied in the analysis of both MC and LTC insurance data, and a cross-sectional design was used for the questionnaire survey. The study was conducted in city A, Hokkaido, Japan. The name of City A cannot be disclosed because the city administration and the ethics committee restricted sharing the city name in order to protect participant privacy. City A is located near the center of the northern island of Hokkaido and has an inland climate. The average annual temperature is 5.5°C, with a mean annual rainfall of 960 mm and snowfall of 884 cm (data from 1980 to 2010) [17]. The inclusion criteria for participation in the study are follows: being 65 years of age, or older; being eligible for Japanese LTC insurance and utilizing LTC services at home or in an institutional setting in December 2009; and being covered by the NHI system and/or MC insurance system.

In the Japanese LTC insurance system, eligibility is classified into seven levels, depending on an individual’s current cognitive, physical, and mental status. The seven levels are as follows: Support Levels 1 and 2, in which individuals require partial assistance in daily living and can access preventative care services in the LTC insurance system (including physiotherapy, nutritional support, and dental care); and Care Needs Levels 1–5. The latter group ranges from those in Level 1, classified as needing partial care for daily life, to Level 5, in which individuals are unable to conduct daily activities without extensive assistance. In order to determine the eligibility level, a trained local government official visits the home and/or institution. Then, he/she uses a questionnaire, created by the Ministry of Health, Labour, and Welfare, to evaluate the individual’s current physical and mental status. Next, a local expert committee, consisting of medical and care professionals, reviews and evaluates the results and assigns each person to an appropriate level [2]. The level to which people are assigned determines the type of LTC service they receive, as well as the fee for each service in the LTC insurance system.

### Data source

MC insurance data were obtained from claim records of the NHI and/or MC insurance systems for the elderly aged 65 years or older in July and December of 2007, 2008, and 2009 (i.e., a total of six survey points). In order to preserve the anonymity of the participants, the staff of city A deleted all their personal information after handing it over to the researchers. MC claim records include hospital services, outpatient services, pharmacist services, and home medical care, as well as treatments provided by judo-orthopedists, masseuses, chiropractors, and acupuncturists. LTC insurance data from claim records were also obtained for the same period. LTC claim records include in-home care services, community-based services, and facility care services. In this study, the sum of hospital service fees, outpatient service fees, and pharmacist service fees is used as MC costs. The sum of in-home care services, community-based care costs, and facility care services fee is used as LTC costs. Because neither of the sets of claim records have any data on household composition, to obtain such information (on population characteristics), we formulated our own cross-sectional questionnaire. We then distributed this questionnaire to care staff who visited homes or who visited people in institutions in 2009 (n = 871). Our questionnaire includes questions on the number of people covered by LTC insurance and on household composition. We obtained permission from all LTC service companies in city A to conduct the questionnaire survey. Then, we delivered the questionnaire to the LTC service companies, which included eight in-home care companies, three group home companies, and four facility companies. For people living in institutions, staff members were instructed to report on the household component using data on participants prior to them entering the institution. For subjects who lived at home, home care staff members were instructed to gather information at the time of their visit. Then, each company returned their questionnaires to us. Panel data, per subject, were created from the claim records for both MC and LTC. The MC claim records include information on age, gender, illness, prescribed medication (including cost), and monthly hospital and/or outpatient care costs. LTC insurance records contain information on age, gender, eligibility level, types of services needed, income level, and the monthly costs of care in the home or in a facility.

In Japan, medical fees are decided based on the report of the Central Social Insurance Medical Council. In principle, revisions are made once every two years. The medical fee system is a points system. MC costs are calculated as the value of medical fees multiplied by 10 yen. In the revision of 2008, an overall review resulted in no increase in the fees for specific medical services. LTC fees are determined by the Minister of Health, Labour and Welfare after conferring with the Social Security Council. LTC fees are revised every three years and are also based on a points system. LTC costs are calculated as the value of LTC fees multiplied by 10 yen. In the revision of 2009, all fees for LTC insurance services increased in order to increase the salaries of LTC staff. The total remuneration for MC costs was reduced by 0.82%. The LTC insurance system was further revised in 2009, and the total remuneration for LTC costs increased by 3%. Thus, in order to account for the effect of the changes in MC and LTC insurance reimbursements, we multiplied MC costs for 2007 by 0.9918 (1–0.82%) and LTC costs for 2007 and 2008 by 1.03 (1 + 3%).

For LTC insurance, income levels are classified from Level 1 to Level 6. People who receive welfare or a welfare pension and who are not required to pay local resident taxes are assigned to Level 1 or Level 2. People who are members of households that are exempt from municipal taxation are assigned to Level 3. People exempt from municipal taxation are assigned to Level 4, and those who pay municipal taxes, but have a total annual income of less than 2,500,000 yen are assigned to Level 5. Lastly, people who pay municipal taxes and whose total annual income is 2,500,000 yen or more are assigned to Level 6 [18]. All data on each subject were linked across each of the six survey points.

### Data analysis

Variables potentially associated with HC costs and services were selected on the basis of prior research [5–7, 19–21]. We included the following variables: environment (i.e., the effects of seasons); population characteristics (i.e., age, sex, and income level (Levels 1–6)); LTC eligibility level (i.e., Support Level 1 or 2, Care Needs Levels 1–5); household composition (living alone or with others); MC cost; and LTC cost. We found few participants in income level 2, and so combined levels 1 and 2. Therefore, we divided income into five levels. Similarly, few participants required Support Levels 1 and 2 and, therefore, the two levels were combined. As a result, our data contain six eligibility levels. We assumed that the subjects’ household composition did not change during the period of investigation. Finally, we applied the data on household composition contained in the results of the cross-sectional questionnaire in 2009 to those in 2007 and 2008.

Data were analyzed using the t-test, chi-square test, Wilcoxon signed-rank test, and median test to compare population characteristics and HC costs at the different survey points. The distribution of MC was censored at zero for a large portion of the sample. To examine household composition and seasonal effects, we estimated a regression equation with a censored dependent variable using a Tobit model [22]. In the model, the HC cost variable served as the dependent variable, while household composition, seasonal effects, an interaction term (household composition × seasonal effects), age, sex, income level, eligibility level, and year (2007, 2008, 2009) were the independent variables. Year was introduced as a dummy variable and used to adjust the Tobit model. Age was treated as a continuous variable, while sex, income level, eligibility, and year were categorical variables. In our analysis, HC costs are treated on a per-member-per-month basis. All statistical analyses were performed using JMP version 9.0 (SAS Institute Inc., CA, USA) or Stata version 11.0 (Light Stone Inc., TX, USA). A two-tailed p-value of less than 0.05 was considered statistically significant.

The study was approved by the Ethics Committee of the University of Tokyo (approved number 1790). This is a joint study between city A and the researchers, and is intended for use in municipal administration. For the study, we obtained comprehensive agreement from residents, including a notice posted in the office of city A. This notice included information about our study’s aim, the right to refuse participation in the research, the right to withdraw consent, and how we protect personal information. Furthermore, the research purpose was also discussed when staff members explained the questionnaire to participants.

## Results

### Survey area

City A has a population of 31,000. In Japan, local cities, towns, and villages with populations under 50,000 comprise over two-thirds of all municipalities. In 2009, the elderly aged over 65 years accounted for 25.0% of the total population of city A, which was higher than the national average of 22.7%. However, the per capita MC and LTC expenditures for the elderly were similar to the national levels.

### Subject characteristics and total monthly HC costs

Of the 871 cross-sectional questionnaires we distributed, 810 were completed, giving a response rate of 93.0%. Of the 61 that were excluded from the analysis, 44 did not return the questionnaire and 17 did not fill in all required items. Table 1 compares the characteristics of the sample subjects and the excluded subjects using the LTC insurance claim data. There were significantly more non-responders in the lower eligibility levels than in the higher levels. There were also more in home care settings than in institutions (p = 0.001). Consequently, 810 subjects and 4061 observations were used in the final analysis.

**Table 1:** Comparison of sample subjects and excluded subjects.

	Sample subjects	Excluded subjects	
Factors	(n = 810)	(n = 61)	p-value
Age ^a^ mean ± SD	84.4 ± 7.4	82.1 ± 7.1	0.022
Sex ^b^ n (%)			
Female	570 (70.4)	43 (70.5)	0.984
Male	240 (29.6)	18 (29.5)	
Income levels ^c^ n (%)			
Level 1/2	385 (47.5)	24 (39.3)	0.233
Level 3	270 (33.3)	19 (31.1)	
Level 4	51 (6.3)	8 (13.1)	
Level 5	77 (9.5)	7 (11.5)	
Level 6	27 (3.3)	1 (1.6)	
Care needs level ^c^ n (%)			
Support Level 1/2	203 (25.1)	27 (44.3)	0.001
Care Level 1	142 (17.5)	7 (11.5)	
Care Level 2	164 (20.2)	13 (21.3)	
Care Level 3	150 (18.5)	4 (6.6)	
Care Level 4	85 (10.5)	6 (9.8)	
Care Level 5	66 (8.1)	4 (6.6)	
Care setting ^b^^,^ ^d^ n (%)			
Home	539 (66.5)	50 (82.0)	0.001
Residential facility	30 (3.7)	0 (0.0)	
LTC facility	233 (28.8)	7 (6.6)	
Hospital	8(1.0)	4(6.6)	

MC, medical care; LTC, long-term care; SD, standard deviation

p-values were calculated using a

^a^t-test,

^b^chi-squared test, or

^c^Wilcoxon rank test.

^d^ Care setting refers to a place where a person spends more than 15 days in a month, with one month being 30 days

Tables 2–4 report the characteristics of the 810 subjects by year. The mean age was 84 years, 30% were male, and many were in income levels 1 and 2 (48%) and in Support Levels 1 and 2 (25%) in the LTC insurance system. The eligibility level in this study was similar to the national level [23]. Of the respondents, 68% were cared for in their own home. Individuals living alone were more likely to be categorized in the lower income group and as being eligible for LTC insurance than those who did not live alone. The differences between winter and summer and across years were not statistically significant.

**Table 2:** Characteristics of subjects in 2007 in city A, Japan.

	Summer			Winter		
	Alone	Other		Alone	Other	
Factors	(n = 160)	(n = 377)	p-value	(n = 174)	(n = 405)	p-value
Age ^a^ mean ± SD	81.6 ± 7.0	82.9 ± 7.9	0.074	81.7 ± 6.9	83.2 ± 7.9	0.037
Sex ^b^ n (%)						
Female	126 (78.8)	269 (71.4)	0.087	137 (78.7)	289 (71.4)	0.080
Male	34 (21.3)	108 (28.6)		37 (21.3)	116 (28.6)	
Income levels ^c^ n (%)						
Level 1/2	101 (63.1)	163 (43.2)	< 0.001	105 (60.3)	170 (42.0)	< 0.001
Level 3	35 (21.9)	54 (14.3)		39 (22.4)	62 (15.3)	
Level 4	7 (4.4)	117 (31.0)		11 (6.3)	129 (31.9)	
Level 5	13 (8.1)	33 (8.8)		15 (8.6)	35 (8.6)	
Level 6	4 (2.5)	10 (2.7)		4 (2.3)	9 (2.2)	
Care needs levels ^c^ n (%)						
Support Level 1/2	62 (38.8)	100 (26.5)	< 0.001	71 (40.8)	118 (29.1)	< 0.001
Care Level 1	48 (30.0)	111 (29.4)		42 (24.1)	92 (22.7)	
Care Level 2	24 (15.0)	57 (15.1)		30 (17.2)	74 (18.3)	
Care Level 3	17 (10.6)	55 (14.6)		22 (12.6)	61 (15.1)	
Care Level 4	6 (3.8)	29 (7.7)		4 (2.3)	35 (8.6)	
Care Level 5	3 (1.9)	25 (6.6)		5 (2.9)	25 (6.2)	
Care setting ^b^^,^ ^d^ n (%)						
Home	99 (61.9)	224 (59.4)	0.480	110 (63.2)	247 (61.0)	0.436
Residential facility	7 (4.4)	11 (2.9)		10 (5.7)	11 (2.7)	
LTC facility	54 (33.8)	142 (37.7)		54 (31.0)	147 (36.3)	

MC, medical care; LTC, long-term care; SD, standard deviation

p-values were calculated using a

^a^t-test,

^b^chi-squared test, or

^c^Wilcoxon rank test.

^d^ Care setting refers to a place where a person spends more than 15 days in a month, with one month being 30 days

**Table 3:** Characteristics of subjects in 2008 in city A, Japan.

	Summer			Winter		
	Alone	Other		Alone	Other	
Factors	(n = 200)	(n = 459)	p-value	(n = 213)	(n = 487)	p-value
Age ^a^ mean ± SD	82.5 ± 6.9	83.7 ± 7.8	0.062	82.8 ± 6.9	83.9 ± 7.7	0.064
Sex ^b^ n (%)						
Female	152 (76.0)	318 (69.3)	0.092	162 (76.1)	334 (68.6)	0.047
Male	48 (24.0)	141 (30.7)		51 (23.9)	153 (31.4)	
Income levels ^c^ n (%)						
Level 1/2	123 (61.5)	183 (39.9)	< 0.001	132 (62.0)	191 (39.2)	< 0.001
Level 3	44 (22.0)	70 (15.3)		46 (21.6)	72 (14.8)	
Level 4	10 (5.0)	150 (32.7)		11 (5.2)	159 (32.6)	
Level 5	17 (8.5)	41 (8.9)		18 (8.5)	50 (10.3)	
Level 6	6 (3.0)	15 (3.3)		6 (2.8)	15 (3.1)	
Care needs levels ^c^ n (%)						
Support level 1/2	79 (39.5)	129 (28.1)	< 0.001	83 (39.0)	111 (22.8)	< 0.001
Care level 1	39 (19.5)	84 (18.3)		35 (16.4)	95 (19.5)	
Care level 2	40 (20.0)	90 (19.6)		44 (20.7)	101 (20.7)	
Care level 3	27 (13.5)	79 (17.2)		35 (16.4)	81 (16.6)	
Care level 4	6 (3.0)	47 (10.2)		6 (2.8)	62 (12.7)	
Care level 5	9 (4.5)	30 (6.5)		10 (4.7)	37 (7.6)	
Care setting ^b^^,^ ^d^ n (%)						
Home	126 (63.0)	286 (62.3)	0.561	137 (64.3)	311 (63.9)	0.624
Residential facility	13 (6.5)	15 (3.3)		13 (6.1)	16 (3.3)	
LTC facility	61 (30.5)	158 (34.4)		63 (29.6)	160 (32.9)	

MC, medical care; LTC, long-term care; SD, standard deviation

p-values were calculated using a

^a^t-test,

^b^chi-squared test, or

^c^Wilcoxon rank test.

^d^ Care setting refers to a place where a person spends more than 15 days in a month, with one month being 30 days

**Table 4:** Characteristics of subjects in 2009 in city A, Japan.

	Summer			Winter		
	Alone	Other		Alone	Other	
Factors	(n = 231)	(n = 545)	p-value	(n = 243)	(n = 567)	p-value
Age ^a^ mean ± SD	83.4 ± 6.7	84.4 ± 7.6	0.077	83.6 ± 6.7	84.7 ± 7.6	0.042
Sex ^b^ n (%)						
Female	178 (77.1)	371 (68.1)	0.012	187 (77.0)	384 (67.7)	0.009
Male	53 (22.9)	174 (31.9)		56 (23.0)	183 (32.3)	
Income levels ^c^ n (%)						
Level 1/2	145 (62.8)	225 (41.3)	< 0.001	153 (63.0)	232 (40.9)	< 0.001
Level 3	57 (24.7)	200 (36.7)		61 (25.1)	209 (36.9)	
Level 4	4 (1.7)	44 (8.1)		4 (1.6)	47 (8.3)	
Level 5	17 (7.4)	57 (10.5)		17 (7.0)	60 (10.6)	
Level 6	8 (3.5)	19 (3.5)		8 (3.3)	19 (3.4)	
Care needs levels ^c^ n (%)						
Support Level 1/2	83 (35.9)	120 (22.0)	< 0.001	83 (34.2)	120 (21.2)	< 0.001
Care Level 1	43 (18.6)	102 (18.7)		40 (16.5)	102 (18.0)	
Care Level 2	44 (19.0)	115 (21.1)		50 (20.6)	114 (20.1)	
Care Level 3	42 (18.2)	96 (17.6)		46 (18.9)	104 (18.3)	
Care Level 4	10 (4.3)	67 (12.3)		13 (5.3)	72 (12.7)	
Care Level 5	9 (3.9)	45 (8.3)		11 (4.5)	55 (9.7)	
Care setting ^b^^,^ ^d^ n (%)						
Home	153 (66.2)	368 (67.5)	0.933	163 (67.1)	389 (68.6)	0.857
Residential facility	14 (6.1)	16 (2.9)		14 (5.8)	16 (2.8)	
LTC facility	64 (27.7)	161 (29.5)		66 (27.2)	162 (28.6)	

MC, medical care; LTC, long-term care; SD, standard deviation

p-values were calculated using a

^a^t-test,

^b^chi-squared test, or

^c^Wilcoxon rank test.

^d^ Care setting refers to a place where a person spends more than 15 days in a month, with one month being 30 days

Tables 5–7 present the HC costs. There were no statistically significant differences in MC and LTC costs throughout the examination period between individuals living alone and those living with others. The median values of costs varied between USD 271 and USD 384 and between USD 682 and USD 1083 for MC and LTC, respectively. The differences between winter and summer and across years were not statistically significant.

**Table 5:** HC costs of subjects for both MC and LTC costs in 2007 in city A, Japan.

	Summer			Winter		
	Alone	Others		Alone	Others	
Factors	(n = 160)	(n = 377)	p-value	(n = 174)	(n = 405)	p-value
MC cost (USD)						
Median	344.8	312.9	0.332	295.0	271.1	0.568
Minimum	0.0	0.0		0.0	0.0	
Maximum	8285.9	12512.1		14314.9	6537.9	
LTC cost (USD)						
Median	681.8	769.5	0.591	684.3	753.4	0.738
Minimum	0.0	0.0		0.0	0.0	
Maximum	3698.4	4638.6		4144.2	3934.0	

HC, healthcare; MC, medical care; LTC, long-term care

p-values were calculated using a t-test.

USD/person/month, USD 1 = JPY 93.5 in 2009

**Table 6:** HC costs of subjects for both MC and LTC costs in 2008 in city A, Japan.

	Summer			Winter		
	Alone	Others		Alone	Others	
Factors	(n = 200)	(n = 459)	p-value	(n = 213)	(n = 487)	p-value
MC cost (USD)						
Median	383.5	299.6	0.059	344.2	313.9	0.460
Minimum	0.0	0.0		0.0	0.0	
Maximum	8849.6	26961.9		9038.0	25796.7	
LTC cost (USD)						
Median	806.3	863.5	0.754	907.9	893.8	0.935
Minimum	0.0	0.0		0.0	0.0	
Maximum	4400.0	4519.2		3913.6	4035.6	

HC, healthcare; MC, medical care; LTC, long-term care

p-values were calculated using a t-test.

USD/person/month, USD 1 = JPY 93.5 in 2009

**Table 7:** HC costs of subjects for both MC and LTC costs in 2009 in city A, Japan.

	Summer			Winter		
	Alone	Others		Alone	Others	
Factors	(n = 231)	(n = 545)	p-value	(n = 243)	(n = 567)	p-value
MC cost (USD)						
Median	370.7	326.3	0.308	355.9	352.3	0.939
Minimum	0.0	0.0		0.0	0.0	
Maximum	8278.6	13115.4		5998.5	8015.3	
LTC cost (USD)						
Median	930.5	971.7	0.814	1083.3	1034.0	0.939
Minimum	0.0	0.0		76.1	31.1	
Maximum	3895.7	3995.2		3998.5	3905.3	

HC, healthcare; MC, medical care; LTC, long-term care

p-values were calculated using a t-test.

USD/person/month, USD 1 = JPY 93.5 in 2009

### Tobit analysis: Effects of the interaction term on HC costs

Table 8 reports the results of the Tobit analysis, which identifies the effects of household composition and season on HC costs. Winter and the interaction term (living alone × winter) had significant negative effects on MC costs, with p = 0.016 and p = 0.020, respectively. Monthly MC costs during winter were USD 134 lower than they were during summer. Monthly MC costs for subjects who lived alone during winter were USD 357 lower than for those living with other household members during summer. Living alone and the interaction term (living alone × winter) had significant positive effects on LTC costs, with p ≤ 0.001 and p ≤ 0.001, respectively. In particular, the monthly LTC costs for subjects living alone were USD 586 higher in winter compared to those living with others in summer. Income level was a positive determinant of MC costs. Those at Level 6 had MC costs that were USD 615 higher than those at Level 1/Level 2 (p = 0.002). However, the LTC costs of those at Level 6 were USD 397 lower than those at Level 1/Level 2 (p < 0.001). The eligibility levels were also strong positive factors in determining LTC costs. Those at Care Level 5 had LTC costs that were USD 2409 higher than those at LTC Support Levels 1/2 (p < 0.001).

**Table 8:** Tobit analysis: Effects of interaction terms on HC costs of subjects in city A, Japan.

		MC cost		LTC cost	
Factors		Coefficient	p-value	Coefficient	p-value
Living alone (ref. Others)		-69.71	0.476	260.60	< 0.001
Winter (ref. Summer)		-133.93	0.016	25.66	0.218
Alone × Winter (ref. Others)		-223.38	0.020	325.44	< 0.001
Age		-0.03	0.996	17.42	< 0.001
Sex (ref. Male)		-90.54	0.318	-67.87	0.314
Income levels (ref. Level 1/2)	Level 3	205.28	0.018	-222.61	< 0.001
	Level 4	179.67	0.055	-296.63	< 0.001
	Level 5	193.64	0.137	-303.58	< 0.001
	Level 6	614.79	0.002	-396.52	< 0.001
Care needs levels (ref. Support Level 1/2)	Care Level 1	-109.55	0.211	560.65	< 0.001
	Care Level 2	-161.46	0.079	999.03	< 0.001
	Care Level 3	-38.59	0.693	1630.16	< 0.001
	Care Level 4	-195.71	0.113	2207.03	< 0.001
	Care Level 5	134.37	0.347	2409.31	< 0.001

HC, healthcare; MC, medical care; LTC, long-term care; ref, reference

p-values were calculated using the Tobit model.

USD/person/month, USD 1 = JPY 93.5 in 2009

## Discussion

This study examines the effects of household composition and season on HC costs among the elderly enrolled in the LTC insurance system in city A, Japan. When considering factors that influence HC costs paid by the MC and LTC insurance systems, it is necessary to collect a variety of data from both claim records in a single panel for each person. In general, it is difficult to connect claim records for MC and LTC in Japan, which is why few studies have analyzed data from both. However, city A cooperated with our research and allowed us to connect the data from claim records for MC and LTC insurances. Thus, we were able to collect various data in a single panel for each subject from both sets of records, which were supplemented by the information from our cross-sectional questionnaires. Using a panel approach, we were able to analyze the impacts of both MC and LTC costs. Our results presented three major conclusions.

First, the household composition variable alone did not have an effect, but the interaction term between household composition and season was negatively associated with MC costs. Seasonal changes affect the mortality risk and the level of MC expenditure of older people [13]. However, it is possible that hospital costs decreased owing to the death of inpatients. Previous studies have reported that the physical activity of an elderly individual is influenced by meteorological factors, particularly precipitation and mean ambient temperature [24, 25]. For elderly individuals living in a large family, family members can take them to a hospital, but for those living alone, such a trip is very difficult when there is heavy snow. Consequently, decreased outpatient care may reduce MC costs for the elderly living alone during winter. If a person requiring an outpatient visit is not able to attend a hospital, their health may suffer as a result. It is necessary to devise a way to protect the access rights of such people, for example, by using a bus system. We did not conduct an analysis by hospital or outpatient services owing to constraints on the sample size. In order to determine whether the decrease in medical expenses in winter is due to hospital services, outpatient services, or pharmacist fees, it is necessary to increase the number of subjects and analyze the types of medical services.

Second, LTC cost was strongly associated with the interaction term between household and season. Our results emphasize that living alone is risk factor for nursing home admission [26]. Those who have severe cognitive or physical disabilities and those who do not have caregivers need to be given preferential admission to nursing homes. The LTC costs for persons living alone were higher than for those living with others. Rolden et al. [13] report that institutionalization rates peak in the winter. We found no effect using the seasonal variable alone, but when the household composition variable was added, the effects became evident. Because nursing homes are almost always full, the changes by season may be small. However, institutionalization may also be progressing, centering on the person living alone. During winter, because it is difficult to go out in the snow, the elderly need help from others to, for example, go shopping, shave, or visit a hospital. Delivery services, such as those for meals, and volunteers who clear snow make it is possible to prevent admissions to facilities for people living alone. More than half the LTC costs are facility costs. Our results do not indicate whether there is an increase in in-home LTC care costs, LTC facility costs, or both in-home care costs and facility costs. There may be a substitutionary effect between MC and LTC among those who live alone during winter, as suggested by the statistically significant negative and positive coefficient estimates, respectively, in the Tobit model. If a person living at home enters an LTC facility, in-home care costs and outpatient treatment fees/pharmacist fees of MC costs decrease. Since per capita facility costs are more than in-home care costs, LTC costs increase significantly. If a person who is hospitalized enters an LTC facility, hospital costs decrease and facility costs increase. These two costs are comparable if the person does not receive specialized in-hospital care, such as an operation. Thus, the relative change in HC will not be substantial. Policymakers need to consider maintaining the health and safety of the elderly living alone during winter.

Third, income level and eligibility level were significantly associated with HC costs. The urban elderly with higher income or their spouses tend to pay for services by themselves or their spouses [12]. It is particularly notable that our results unequivocally indicate that LTC costs are strongly associated with income levels. Akiyama, et al. reported that the subjects with low income has higher facility entry rate than those subjects with higher income [27]. The OECD has reported that living arrangements and income level play an important role in the social protection for individuals of retirement age [28]. Elderly persons living alone are 2.5 times more likely to be poor than are elderly couples, and in most countries, the poverty rate is higher for women over 75 [29]. An elderly household that initially comprises a couple is likely to become a one-person household, because the average female life expectancy is longer than that of males in Japan. The Japanese Ministry of Health estimates that the annual LTC will more than double by 2026, at USD 212 billion, from the March 2013 level as the number of one-person households increases [30]. Low-income elderly living alone may be severely at risk of not receiving the requisite MC and/or LTC. In general, and in our results, an elderly person’s cognitive level is reflected in the LTC insurance eligibility level. The more dependent the elderly are, the more their LTC costs increase. This study found no significant association between eligibility levels and MC costs. Studies have reported that dementia represents a substantial financial burden on society, not only with respect to money, but also in terms of informal caregivers [31].

## Limitation and implications

The present study has several limitations. First, the analysis of factors correlating HC costs only consider a single city in Hokkaido and use data for two months in a year. This creates a selection bias, which is why other studies have found a different trend in the expenditure of rural and urban areas [32]. According to our survey, the average age of the subjects exceeds 80 years, which indicates an aging society. In general, it is possible to live without assistance until the age of 75, but after the age of 80, many people need some kind of care. This survey covers individuals using LTC insurance services with increased LTC costs; thus, the average age is high. In many developed countries, the average life expectancy is over 80 years. Seasonal influences may be found in such countries for the elderly who need LTC. Second, we do not include services such as judo-orthopedists, nursing visits covered by MC insurance, and community-based services. However, these services form a small percentage of all services and, thus, have little impact. Because the number of subjects was small, we could not conduct a comparative analysis between service types, for example, between in-home care and facility care. Third, in our cross-sectional survey, among those excluded from the analysis, there were many users of home services. In a survey targeting the elderly, it is difficult to obtain a response unless it is in the form of an interview. Furthermore, it is challenging for in-home care staff members to go to a subject’s home to obtain confirmation. Thus, there was a high barrier to useful information on household composition in city A. However, household composition is essential when considering LTC policy. Finally, we assumed that household composition did not change during the study period. Moreover, the percentage of persons living alone in this study was 30%, which is slightly higher than the national average of 26% [3]. Future studies should examine this point in regions with different climate variables, such as amount of snow, outside temperature, and road conditions, and should collect data for 12 months and over a long period. Furthermore, a larger sample is required to clarify why MC and LTC costs change by season and by household composition.

Despite these limitations, our results are meaningful and contribute to the identification of factors that influence HC costs in Japan. We strongly recommend that health policies for the elderly focus on the effects of household composition, season, income level, and LTC eligibility.

## Conclusion

This study found that, in addition to income level and eligibility level, household composition and season correlate HC services for the elderly enrolled in the LTC insurance system. There may be a substitutionary effect between MC and LTC among those who live alone and during winter, as suggested by the statistically significant negative and positive coefficient estimates of the Tobit model. MC costs were not correlated by the household variable alone, but were affected by the interaction term of the household and seasonal variables. MC costs decreased significantly. On the other hand, LTC costs were not correlated by the seasonal variable alone, but they increased significantly in the case of the interaction variable. Most elderly persons who use LTC services incur both MC costs and LTC costs. Lastly, the effects of the environment and population characteristics on MC costs and LTC costs differ. In considering health policies for the elderly, it is very important to connect data on MC and LTC insurance, creating one set of panel data.
